# Dual African Origins of Global *Aedes aegypti* s.l. Populations Revealed by Mitochondrial DNA

**DOI:** 10.1371/journal.pntd.0002175

**Published:** 2013-04-18

**Authors:** Michelle Moore, Massamba Sylla, Laura Goss, Marion Warigia Burugu, Rosemary Sang, Luna W. Kamau, Eucharia Unoma Kenya, Chris Bosio, Maria de Lourdes Munoz, Maria Sharakova, William Cormack Black

**Affiliations:** 1 Department of Microbiology, Immunology and Pathology, Colorado State University, Fort Collins, Colorado, United States of America; 2 Centre for Virus Research, Kenya Medical Research Institute, Nairobi, Kenya; 3 Centre for Biotechnology Research and Development, Kenya Medical Research Institute, Nairobi, Kenya; 4 Kenyatta University, Department of Biochemistry, Nairobi, Kenya; 5 Rocky Mountain Laboratories, Hamilton, Montana, United States of America; 6 Department of Genetics and Molecular Biology, Centro de Investigación y de Estudios Avanzados del Instituto Politécnico Nacional, San Pedro Zacatenco, Gustavo A. Madero, México D. F., México; 7 Fralin Biotechnology Center, Virginia Tech, Blacksburg, Virginia, United States of America; Centers for Disease Control and Prevention, United States of America

## Abstract

**Background:**

*Aedes aegypti* is the primary global vector to humans of yellow fever and dengue flaviviruses. Over the past 50 years, many population genetic studies have documented large genetic differences among global populations of this species. These studies initially used morphological polymorphisms, followed later by allozymes, and most recently various molecular genetic markers including microsatellites and mitochondrial markers. In particular, since 2000, fourteen publications and four unpublished datasets have used sequence data from the NADH dehydrogenase subunit 4 mitochondrial gene to compare *Ae. aegypti* collections and collectively 95 unique mtDNA haplotypes have been found. Phylogenetic analyses in these many studies consistently resolved two clades but no comprehensive study of mtDNA haplotypes have been made in Africa, the continent in which the species originated.

**Methods and Findings:**

ND4 haplotypes were sequenced in 426 *Ae. aegypti* s.l. from Senegal, West Africa and Kenya, East Africa. In Senegal 15 and in Kenya 7 new haplotypes were discovered. When added to the 95 published haplotypes and including 6 African *Aedes* species as outgroups, phylogenetic analyses showed that all but one Senegal haplotype occurred in a basal clade while most East African haplotypes occurred in a second clade arising from the basal clade. Globally distributed haplotypes occurred in both clades demonstrating that populations outside Africa consist of mixtures of mosquitoes from both clades.

**Conclusions:**

Populations of *Ae. aegypti* outside Africa consist of mosquitoes arising from one of two ancestral clades. One clade is basal and primarily associated with West Africa while the second arises from the first and contains primarily mosquitoes from East Africa

## Author Summary

The authors are all medical entomologists who have worked in the field for more than 30 years. Over the past 20 years we have primarily worked on *Aedes aegypti*, the primary mosquito vector of Dengue and Yellow Fever Viruses. Twelve years ago, we began using mitochondrial markers to study relationships among *Ae. aegypti* populations. Since that time, 14 publications and 4 datasets have used the same markers and collectively 95 unique mtDNA haplotypes have been found. Haplotype phylogenies have consistently identified two clades. However, it wasn't until we combined efforts with our African colleagues that we realized that the two clades largely correspond with West and East Africa. *Aedes aegypti* populations from throughout the world are “mixtures” of mosquitoes from these two original clades. We plan to continue this effort to determine whether the composition of *Ae. aegypti* populations affects their ability to transmit arboviruses and also if mitochondrial haplotypes differ between mosquitoes with or without the newly discovered chromosomal inversions.

## Introduction

*Aedes aegypti*, the ‘yellow fever mosquito’, is the primary vector to humans of the four serotypes of dengue flaviviruses (DENV1-4) and the yellow fever flavivirus (YFV). Dengue is a major public health problem in the tropics, causing millions of dengue fever and hundreds of thousands of dengue hemorrhagic fever cases annually [1]. In endemic areas the annual number of cases has risen steeply since the 1950s [2]. With multiple serotypes circulating in endemic areas, 100 million infections of dengue fever (DF) occur annually, including up to 500,000 cases of the more severe form of disease called dengue hemorrhagic fever (DHF) with a case fatality rate of up to 5% [3]. Despite the development of a safe, effective YFV vaccine, yellow fever remains an important health risk in sub-Saharan Africa and tropical South America [4], [5]. The WHO estimates that there are 200,000 cases and 30,000 deaths attributable to YFV infection each year, most of which occur in Africa [6].

There are two recognized subspecies of *Ae. aegypti* s.l., the presumed ancestral form, *Ae. aegypti formosus (Aaf)*, a sylvan mosquito supposedly limited to sub-Saharan Africa; and *Ae. aegypti aegypti (Aaa)*, found globally in tropical and subtropical regions typically in association with humans. The designation of *Ae. aegypti* s.l. subspecies arose from observations made in East Africa in the late 1950's that the frequency of pale “forms” of *Ae. aegypti* was higher in populations in and around human dwellings than in adjacent forests [7], [8]. The implied correlation between color and behavior prompted Mattingly to revisit the biology and taxonomy of *Ae. aegypti* [9]. He described *formosus* (Walker) as a subspecies of *Ae. aegypti* that was restricted to sub-Saharan Africa and in West Africa “is the only form known to occur except in coastal districts and in one or two areas of limited island penetration.” However, this latter statement was based only on two collections, one from Ghana and the other from Burkina Faso. He also suggested that *Aaf* most frequently breeds in natural containers such as tree holes, and feeds primarily on wild animals. Mattingly also stated that in addition to the dark-scaled parts of the body being generally blacker, “*ssp. formosus* never has any scales on the first abdominal tergite.” The type form of *Aaa* was alternatively defined as “either distinctly paler and browner (at least in the female) than *ssp. formosus* or with pale scaling on the first abdominal tergite or both.” He also suggested that *Aaa* breeds in artificial containers provided by humans, will breed indoors, and has a preference for feeding on human blood [9].

The subsequent studies of Tabachnick, Powell, Munstermann and Wallis [10]–[21] on the population genetics and vector competence of *Ae. aegypti* s.l. showed that global collections fell into two clades. One clade contained *Aaa* from East Africa, South America and the Caribbean suggesting that these New World populations were derived from East Africa. The other clade contained Asian and Southeastern U.S. *Aaa* and a basal branch containing *Aaf* from both East and West Africa suggesting an independent New World and Asian introduction. Their parallel work on vector competence [11]–[13] showed that West African *Aaf* had lower competence for YFV than other global collections of *Aaf* and *Aaa*. A more recent study examined 24 worldwide collections of *Ae. aegypti* s.l. at 12 polymorphic microsatellite loci [22]. Two distinct genetic clusters were identified: one included all domestic populations outside of Africa and the other included both domestic and forest populations within Africa.

Fourteen papers published since 2000 [23]–[37] and 4 unpublished datasets on GenBank used sequence variation in the mitochondrial NADH dehydrogenase subunit 4 (ND4) gene to describe patterns of gene flow among *Ae. aegypti* s.l. collections within and among countries outside Africa (Table 1). For example, the first paper in Table 1 was a population genetic analysis of gene flow among 10 *Aedes aegypti* collections from seven cities along the northeastern coast of Mexico [23]. A total of 574 mosquitoes were examined and 9 novel ND4 haplotypes were discovered. Using Tamura-Nei distance [38] and neighbor joining [39], haplotypes were placed into two clades with 90% and 99% support. Table 1 documents that to date 95 novel ND4 haplotypes have been discovered and that two phylogenetic patterns were consistently noted: either mtDNA haplotypes were distributed on two well supported clades (pattern 1), seen in three published datasets [23], [25], [35], and three unpublished datasets (GB1, GB2, GB4) or as a basal group (more similar to the outgroup species) from which a second well supported derived (less similar to the outgroup species) clade arises (pattern 2 - publications [24], [28], [30], [32]–[34], [36]. These patterns are not limited to the mitochondrial ND4 gene. A study in Brazil utilized the mitochondrial Cytochrome Oxidase I (COI) gene to examine gene flow among 163 mosquitoes in 14 collections [40]. Their phylogenetic analysis identified two clades with 81 and 96% bootstrap support. Based upon comparison with GenBank COI sequences [41] from an *Ae. aegypti* strain collected from Kenya, another from West Africa and a third *Aaf* strain; they designated one clade as “East African” and the other as “West African.” A study in Argentina that included collections from Brazil, Paraguay, Uruguay and Bolivia utilized Restriction Fragment Length Polymorphism (RFLP) analysis of the ND4, ND5, COI and COII mitochondrial genes and identified three clades [34]. However, since that study did not include sequence data for these four genes they could not be compared to sequences in the present study. A combined study of *Aaa*, *Aaf*, *Ae. albopictus* and *Ae. mascarensis* from islands in the southwest Indian Ocean examined phylogenetic relationships within and among all four taxa [41]. Bayesian phylogenetic analysis clearly differentiated two clades; one (labeled GR1) had a credibility value of 0.81 and contained all mosquitoes identified as *Aaf* while a second clade (GR2) had a credibility value of 0.86 and contained all *Aaa* mosquitoes. *Aaa* and *Aaf* were monophyletic with *Ae. mascarensis* immediately basal. A study using microsatellites and the mitochondrial ND4 and COI genes in Bolivia detected two clades [36] with credibility values of 0.75–0.76.

**Table 1:** Fourteen publications and 3 unpublished (GenBank) databases of *Aedes aegypti sl* mitochondrial ND4 sequences.

Publ.	Year	Number of Locations Sampled	Number of Mosquitoes Sampled	Number of Haplotypes found	Number of New Haplotypes discovered	% Bootstrap Support		Phylogenetic method
						Basal	Derived	
23	2000	10	574	9	9	90	99	TN–NJ^b^
24	2002	38	1977	23	15	<50	86	TN–NJ^b^
25	2005	19	1346	7	6	100	77	TN–NJ^b^
26	2005	3	55	3	1	too few	too few	
GB1	2006^a^	unpubl.	unpubl.	17	6	100	65	TN–NJ^c^
27	2006	24	1144	6	?	?	?	MP^d^
28	2007	42	218	20	12	51	92	MP^d^
29	2007	5	176	4	3	too few	too few	
30	2008	12	125	19	7	<50	94	TN–NJ^c^
31	2008	9	619	6	0	too few	too few	
32	2009	19	654	9	0	<50	86	TN–NJ^b^
33	2009	3	166	38	20	<50	72	TN–NJ^c^
34	2009	25	572	20	?	No seq	No seq	
35	2009	10	123	13	8	77	73	TN–NJ^c^
GB2	2011^a^	unpubl.	unpubl.	2	1	too few	too few	
GB3	2011^a^	unpubl.	unpubl.	8	6	91	89	TN–NJ^c^
36	2012	21	127	4,8^f^	0	0.75^g^	0.76^g^	Bayes^e^
37	2007	1	1	1	1	too few	too few	
**TOTAL**		**241**	**7877**	**209**	**95**			

a – data only appears in GenBank, GB1 = Costa,M.C.V., Paduan,K.S., Ribolla,P.E.M. and Lourenco-de-Oliveira,R., GB2 = Bona,A.C.D., Twerdochlib,A.L., Leandro,A.S., Kafka,R. and Nararro-Silva,M.A. GB3 = Twerdochlib,A.L., Bona,A.C.D. and Navarro-Silva,M.A. GenBank Accession numbers appear in Table S1.

b – Tamura-Nei genetic distance/Neighbor-Joining in the original publication.

c – Tamura-Nei genetic distance/Neighbor-Joining applied in the present study.

d – Maximum Parsimony phylogenetic analyses.

e – Maximum Likelihood/Bayesian phylogenetic analyses.

f – first value is the number of ND4 haplotypes followed by the number of COI haplotypes.

g – clade credibility scores.

Despite the large numbers of studies that have detected these two mitochondrial clades, no studies have been made of the clades in continental Africa. Assumptions about the African origin of *Ae. aegypti* s.l. are based upon the observation that 58 species of the subgenus *Stegomyia* are also endemic to Africa [42] and the greatest genetic diversity in allozymes markers [14], [15], [18] and microsatellites [22] in *Ae. aegypti* s.l. are found in African collections.

It is currently unclear if there is an association between the two well documented mitochondrial clades in the literature and the Aaa and Aaf subspecies or if the clades are differentially associated with East versus West Africa. To address this deficiency, the present study examines ND4 haplotypes among 426 Ae. aegypti s.l. collected at 10 locations in Senegal, West Africa and seven novel haplotypes collected in 7 locations in Kenya in East Africa. A comparison of these sequences was then made with the 95 existing haplotypes detected and reported globally in the literature (Table 1).

## Materials and Methods

### *Aedes aegypti* collections and extraction of DNA

Over three years (2005–2008) *Ae. aegypti* larvae were collected from 10 locations in Senegal (Table 2). These were raised to adults in a field laboratory, bloodfed and eggs were collected. Eggs were transported to Colorado State University where they were hatched and reared to adults. Immediately following eclosion, males and females were classified as either *Aaa* or *Aaf* using McClelland's [7] scale pattern system. Mosquitoes with any white scales on the first abdominal tergite of the adult were designated *Aaa*. If the first abdominal tergite was completely lacking in white scales then the individual was designated *Aaf*. Adults were allowed to mate and oviposit. DNA was then extracted from each individual using the salt extraction protocol [43] suspended in 300 µl of TE buffer (10 mM Tris-HCl and 1 mM EDTA, pH 8.0), and stored at −80°C. The same procedures were followed with F_1_ mosquitoes collected in East Africa (Table 2).

**Table 2:** Location and samples sizes of *Aedes aegypti s.l.* from 10 collections in Senegal and 7 locations in Kenya*.

Collection	N		
West Africa		Latitude (N)	Longitude(W)
Ouakam	44	14°43′25.89″	17°29′20.99″
Joal-Fadiouth	40	14°09′54.48″	16°49′21.72″
Mont Rolland	47	14°55′10.97″	16°59′28.77″
Ziguinchor	48	12°34′47.63″	16°17′02.38″
Sédhiou	45	12°42′16.38″	15°33′22.31″
Koungheul	43	13°58′33.49″	14°48′15.11″
Dienoudiala	45	13°12′52.05″	13°6′43.15″
Niemenike	47	13°0′25.52″	12°32′48.14″
Fongolimbi	43	12°24′44.88″	12°0′41.76″
PK10 Forest	14	12°36′0.09″	12°14′0.25″

East Africa		Latitude (S)	Longitude(E)
Rabai	4	3°55′57.8″	39°34′20.0″
Changombe 1	1	3°54′56.3″	39°35 48.7
Changombe 2	1	3°54′47.9″	39°35′49.0″
Bengo 1:	1	3°56′00.2″	39°34′19.5″
Bengo 2	1	3°55′52.2″	39°34′15.4″
Mbarakani 1	1	3°56′07.0″	39°34′16.3″
Mbarakani 2	1	3°56′10.5″	39°34′21.4″
TOTAL	426		

* Entomological gathering was not done on private land or in private residences.

DNA was also purified from five other species to serve as outgroups: *Ae. (Stegomyia) metallicus* (Edwards) (JX427526), *Ae. (Stegomyia) luteocephalus* (Newstead) (JX427527), *Ae. (Stegomyia) unilineatus* (Theobald) (JX427530), *Ae. (Fredwardius) vittatus* (Bigot) (JX427529), and *Ae. (Zavortinkus) longipalpis* (Grunberg) (JX427528). All were collected near Kedougou, Senegal and identified using four taxonomic keys [42], [44]–[46]. The *Ae. (Stegomyia) albopictus* (Skuse) sequence was EF153761.

### PCR amplification of ND4

Initially degenerate primers were developed for PCR using the only mosquito mtDNA sequences available in 2000 (*An. gambiae*, *An. albimanus*) [23]. These were ND4+ (5′-GTDYAT TTATGATTRCCTAA-3′) and ND4−(5′-CTTCGDCTTCCWADWCG TTC-3′). Although these primers had been used in five prior studies [23]–[25], [31], [32] they failed to amplify any products using template DNA from Senegal *Ae. aegypti*. New primers were designed once the *Ae. aegypti* mitochondrial genome (EU352212) became available. They were ND4sb+ (5′-TTATGATTGCCAAAGGCTCAT-3′), and ND4sb− (5′-CTTCGTCTTCCTATTCGTTC-3′). The new ND4 primers were optimized on a gradient thermal cycler and had an optimal annealing temperature of 52°C. Amplification failures with African template DNA and the ND4+/− primers probably occurred because these primers were degenerate and because the primer annealing site for ND4+ varied in the Senegal mitochondrial genomes. The size of the amplified product was 387 bp.

These new primers were used to amplify ND4 from the 426 mosquitoes shown in Table 2. PCRs were 25 uL in volume and used Commercial GoTaq (BioRad, Hercules, CA). Single Strand Conformation Polymorphism (SSCP) analysis was performed on amplified PCR products to identify unique haplotypes for each location [43]. The sensitivity and specificity of SSCP were evaluated by sequencing at least two PCR products for each perceived unique SSCP pattern. PCR products were purified using minElute PCR purification kits (Qiagen, Valencia, CA). DNA concentration was determined on a Nanodrop spectro-photometer (N-1000) (ThermoFisher Scientific, Wilmington, DE). Purified DNA was loaded onto a 96 well semi-skirt plate with either the forward or reverse ND4 primers for each sample. The plates were then sent to the Colorado State University sequencing facility http://www.pmf.colostate.edu/dna_sequencing.html. PCR products from 92 mosquitoes were sequenced.

### Analysis of ND4 sequences

NUMTS (Nuclear mtDNA) [47] have been previously reported in *Ae. aegypti* [33], [48]. Because true mitochondrial genomes are haploid, NUMTs are most readily identified by scanning sequences for heterozygous sites (double peaks). To detect NUMTs in the present study, forward and reverse trace files were aligned and tested for heterozygotes using Geneious software (http://www.geneious.com/). No heterozygous sites were detected in any sequences gathered in the present study. However, this approach is not definitive because a NUMT may be entirely homozygous. Three NUMTs were found in GenBank sequences (AF203367, AF203368, AF334847) previously submitted from the senior authors' laboratory [23], [24].

Sequences were aligned using ClustalW http://www.genome.jp/tools/clustalw/. Primer sequences were removed from the 5′ and 3′ ends. Aligned sequences were analyzed with RAxML [49] to identify duplicate sequences. A total of 16 unique haplotypes were found among the 92 sequences and 15 of these were new. The published haplotype (DQ176837) [28] appeared 63 times in 92 sequences and was previously found in Guinea, Uganda, and Singapore.

### Phylogenetic relationships and rates of molecular evolution among haplotypes

All phylogenetic analyses employed Maximum Likelihood with bootstrap analysis using RAxML [49]. Bootstrap support was evaluated with 1000 pseudoreplicates to test the consistency of the derived clades. To test the ML phylogeny, a Bayesian analysis of the same dataset was performed using MrBayes3.2 [50]. Trees were drawn using TreeGraph2 [51]. Distance/Neighbor-joining and Maximum Parsimony trees were not derived because most of the datasets had already been subjected to these analyses in the original publications (Table 1).

## Results

### Phylogenetic relationships among African haplotypes

**Figure 1:**
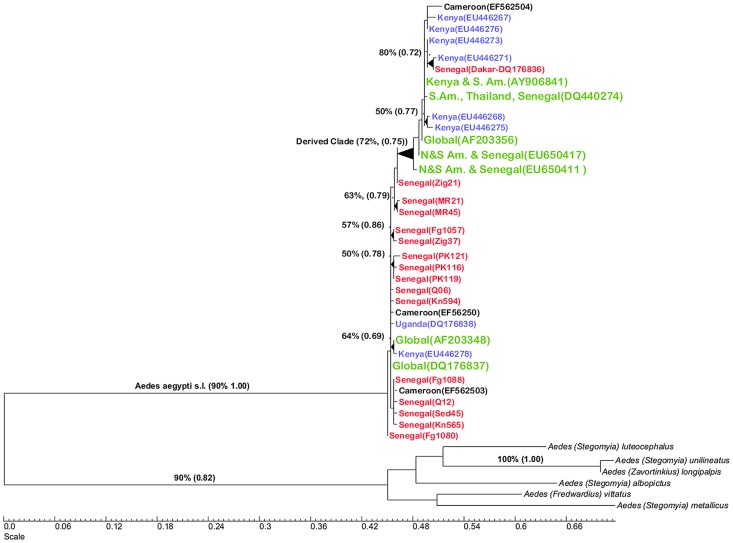
Maximum likelihood tree of the 34 mtDNA ND4 *Ae. aegypti* haplotypes found to date in Africa and outgroups. These were comprised of the 15 new unique Senegal haplotypes from the present study and one Senegal haplotype collected in Dakar in a previous study (labeled in red). Seven novel haplotypes from Kenya and one from Uganda (in blue), three from Cameroon (in black) and seven haplotypes (in large green font) that appeared in collections from Africa and other global locations in various other studies (Table S1). Branches with bootstrap support values >50% are labeled with % support. These support values are followed by clade credibility values in parentheses from MrBayes analysis.

The first dataset analyzed contained the 34 *Ae. aegypti* haplotypes found to date in Africa. These were comprised of the 15 new unique Senegal haplotypes from the present study and one Senegal haplotype collected in Dakar in a previous study [28] (labeled in red in Figure 1). Seven novel haplotypes from Kenya and one from Uganda are labeled in blue, three from Cameroon [29] appear in black and seven haplotypes that appeared in collections from Africa and other global locations in various other studies (Table S1) appear in green. Figure 1 is a ML tree with % bootstrap support and clade credibility scores (*a posteriori* probabilities from Bayesian analysis) appearing over branches with >50% support or credibility scores >0.5.

There are six patterns to note in this phylogeny. First, based upon use as outgroups of four related subgenus *Stegomyia* species and two additional African subgenera, two clades are identified. This clade has a moderate 72% bootstrap support with maximum likelihood analysis and a clade credibility value of 0.75 in the Bayesian analyses. In addition, these same clades were independently detected in seven of the fifteen published studies and in two of the three unpublished GeneBank datasets. One of the clades is basal (more similar to the outgroups) while the second clade is derived (less similar to the outgroups) from the basal clade. Hereafter these are referred to as the “basal” and “derived” clades. Second, all 15 new Senegal haplotypes occur in the basal clade while the one haplotype collected in Dakar belongs to the derived clade. Third, two of the eight east African haplotypes, one from Kenya and one from Uganda appear in the basal clade but six are in the derived clade. Fourth, the basal clade contains two globally distributed haplotypes. AF203348 has been found independently in 9 studies from Mexico, Brazil, Venezuela, Thailand, Tahiti, Cambodia, Singapore, Myanmar, and Kenya (Table S1) while DQ176837 has been found independently in five studies from Guinea, Uganda, Singapore, Cameroon, Brazil, Myanmar, and Senegal. Fifth, the derived clade has three basal branches represented by global haplotypes. EU650411 and EU650417 have been found in Brazil, Senegal, and the USA [35] while AF203356 has been found in 8 studies from Mexico, Brazil, Venezuela, USA, Senegal and Myanmar. Within the derived clade, AY906841 has been collected from Brazil and Kenya while DQ440274 has appeared in 5 studies from Senegal, Venezuela, and Thailand. The sixth pattern is that there was no difference in subspecies composition between the two clades. All Kenyan mosquitoes lacked scales on the first abdominal tergite (were *Aaf*) but occurred on the same clade with mosquitoes previously identified as *Aaa*. Similarly, we have previously shown [52] that *Ae. aegypti* s.l. from northwest Senegal mosquitoes are composed mostly of *Aaa*, while those from southeastern Senegal are mostly pure *Aaf*. Yet all Senegal mosquitoes collected in the present study occur on the basal clade.

### Phylogenetic relationships among all *Ae. aegypti* haplotypes

The results in Fig. 1 prompted us to examine all of the 215 *Ae. aegypti* s.l. ND4 sequences currently on GenBank (Tables 1 & S1). After removing redundant sequences, 95 unique ND4 haplotypes remained. The ML and Bayesian phylogenies containing all 117 (95+15(Senegal) +7(Kenya)) haplotypes and outgroups appear in Figures S1 and S2 respectively. The same six patterns noted in Figure 1 are repeated in these two full analyses. Of the 65 haplotypes that occur in the basal clade, 19 are from Africa (15 from Senegal, 2 from Cameroon and 1 each from Kenya and Uganda), 8 are from North America, 16 are from South America, and 13 are from Southeast Asia. The basal group contains 6 global or widely distributed haplotypes (AF203348, DQ176837 from Fig. 1, DQ176845, DQ176848, and AF203346 from the New World, and EF153747 from the New World and Thailand). The three NUMTs in GenBank appear at the base of the basal group.

Of the 52 haplotypes that occur in the derived clade, 8 are from Africa (1 from Senegal, 1 from Cameroon and 6 from Kenya), 7 are from North America, 15 are from South America, and 13 are from Southeast Asia. The derived group contains 8 global or widely distributed haplotypes (EU650411, EU650417, AF203356, AY906841 and DQ440274 from Fig. 1, AF203344, AF334863, and AF334860 from the New World). No NUMTs were found in the derived clade.

## Discussion

The phylogeny displayed in Figure 1, the phylogenetic analyses of all mtDNA ND4 haplotypes reported to date (Figs. S1 & S2) in addition to the fourteen independently derived phylogenies that appear in publications (Table 1) all support an hypothesis that *Ae. aegypti* populations from around the world consist of mosquitoes that arise from one of two matrilineages. Outgroups consisting of four related subgenus *Stegomyia* species and two additional African subgenera, consistently indicate that one of the *Ae. aegypti* matrilineages is basal while the second matrilineage arises from the first.

The purpose of this study was to trace the African origins of these two clades. Key observations are that all but one of the ND4 haplotypes from Senegal occur on the basal matrilineage whilst haplotypes from East Africa arise predominantly on the second, derived matrilineage (Fig. 1). However, samples from Kenya are only from the Rabaï area. Mbarakani, Bengo and Rabaï are approximately 100 m apart and this cluster is 4 km from Changombe. Further, Rabaï is 14 km inland from Mombassa on the coast. Mombassa is the second largest city of Kenya and a major port. Thus, as with Dakar in Senegal, Mombassa could easily be a place where *Ae. aegypti* immigrate through human commerce. It would be very interesting to sample *Ae. aegypti* from other locations further inland in East Africa to assess this possibility.

This pattern prompted us to re-examine all of the 215 *Ae. aegypti* s.l. ND4 sequences in GenBank (Table S1). Phylogenetic analyses of the 95 unique haplotypes confirmed that all but one West African haplotype occurred on the basal matrilineage. This matrilineage also contained many globally distributed haplotypes. Conversely, most east African haplotypes occurred on the derived matrilineage which also contained many globally distributed haplotypes. The phylogenies presented here demonstrate that *Ae. aegypti* populations outside Africa consist of “mixtures” of mosquitoes from both the basal and derived matrilineages.

Figure 1 is ambiguous as to whether *Aaf* or *Aaa* (*sensu* Mattingly) was the ancestor because basal haplotypes were detected in mosquitoes with and without scales on the first abdominal tergite [52]. This result is not surprising given that McClelland's 1974 study [53] also found collections of almost pure *Aaf* in Pensacola, Key West and Miami, Florida. Conversely, collections from Kenya, Nigeria, Tanzania, Senegal, Ghana, Burkina Faso, Sri Lanka, Calcutta, Jamaica, and Miami Airport contained diverse mixtures of *Aaf* and *Aaa* mosquitoes. Inferences about subspecies composition and West versus East African origins cannot be inferred from the earlier allozymes studies [10]–[21] nor from the recent microsatellite study [22] because they did not use McClelland's [53] scoring scheme nor did they include an outgroup.

The current study is unique in providing the first mitochondrial ND4 data from West Africa and definitively associating the two clades reported in the literature with West and East Africa. We strongly emphasize that the hypotheses and patterns described in this paper are not novel. Bracco et al [28] made 36 collections throughout the New World (Brazil, Peru, Venezuela, Guatemala, US), three from Africa (Guinea, Senegal, Uganda), and three from Asia (Singapore, Cambodia, Tahiti). They also detected two clades and concluded that “three percent of nucleotide divergence between these two clades is suggestive of a gene pool division that may support the hypothesis of occurrence of two subspecies of *Ae. aegypti* in the Americas.” Later the two clades were actually labeled as East and West African albeit based on only three haplotypes from long established laboratory strains [40]. Most recently a combined analysis of ND4 and CO1 also associated one clade (lineage 2) with West Africa [41].

We have recently discovered multiple chromosome inversions in *Ae. aegypti* s.l. [54] and M. Sharakova obtained direct visual evidence with Fluorescent *In Situ* Hybridization (FISH) for inversions on each arm of the third chromosome (unpublished). An obvious question arises as to how these inversions correspond to mosquitoes from the two clades and to the global versus African *Ae. aegypti* microsatellite clades [22].

## Supporting Information

Figure S1 **Maximum likelihood tree of the 117 mtDNA ND4** ***Ae. aegypti*** **haplotypes discovered to date and outgroups.** Haplotypes collected only in Africa appear in red, South America in blue, North America in gold, Southeast Asia in purple and those collected in more than one geographic area in green. All of the NUMTS found in GenBank to date appear as olive drab and are labeled NUMT. Branches with bootstrap support values >50% are labeled with % support. Maximum likelihood branch lengths are proportional to the number of nucleotide substitutions per site. The scale at the bottom of this figure is from 0.0–0.07.
(PDF)

Figure S2 **Bayesian tree of the 117 mtDNA ND4** ***Ae. aegypti*** **haplotypes discovered to date and outgroups.** Haplotypes collected only in Africa appear in red, south America in blue, North America in gold, Southeast Asia in purple and those collected in more than one geographic area in green. Branches with bootstrap support values >50% are labeled with % support.
(PDF)

Table S1 **Unique mitochondrial ND4 sequences in GenBank (117 total) listed according to the order of appearance in the literature or in GenBank.** The third column contains the GenBank # for entries with identical sequences to the initial submission. There are 214 entries (rows) in the table. The 4^th^ column indicates whether the haplotype occurs in the basal (B) or the derived (D) clade. The name of the haplotype as it appears in the original publication (column 7) is listed in the 5^th^ columns and the collection location in the 6^th^ column.
(DOC)
