# Supplementary figures and images for: Dual African Origins of Global Aedes aegypti s.l. Populations Revealed by Mitochondrial DNA
Source: PLoS Negl Trop Dis. 2013 Apr 18;7(4):e2175. doi: 10.1371/journal.pntd.0002175 (PMC3630099; doi:10.1371/journal.pntd.0002175)

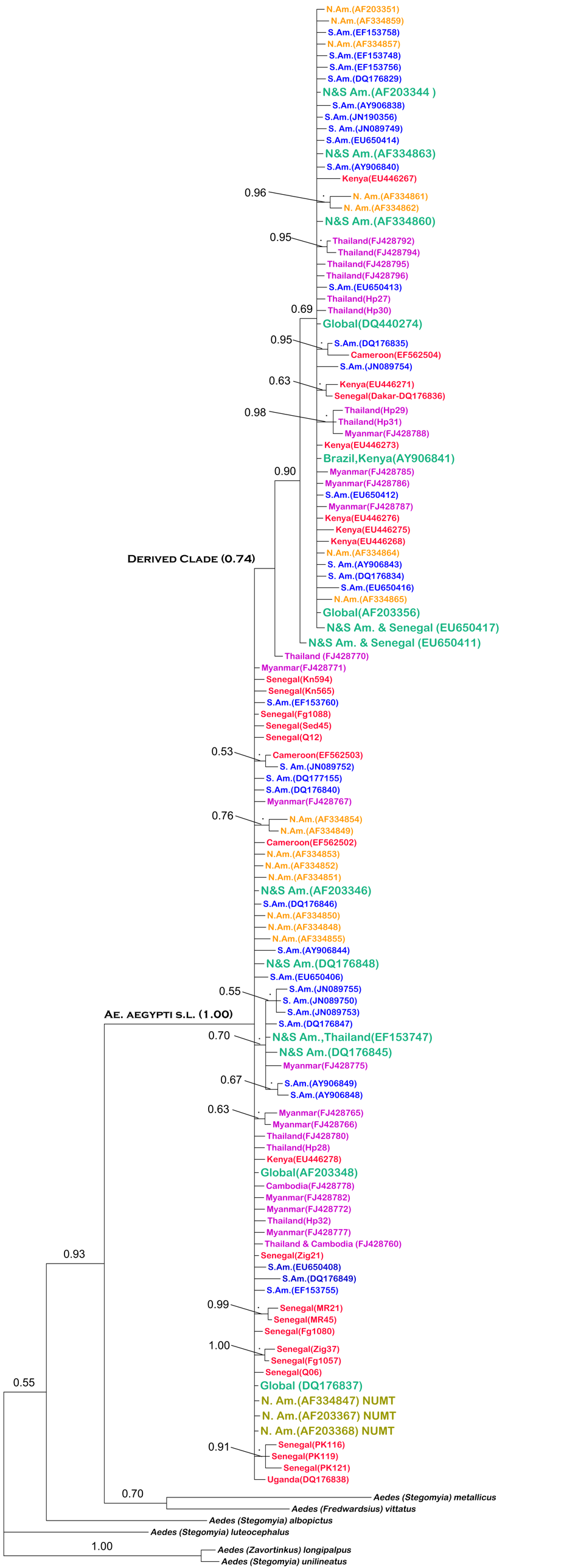

Supplement: Figure S2 — Bayesian tree of the 117 mtDNA ND4 Ae. aegypti haplotypes discovered to date and outgroups. Haplotypes collected only in Africa appear in red, south America in blue, North America in gold, Southeast Asia in purple and those collected in more than one geographic area in green. Branches with bootstrap support values >50% are labeled with % support. (PDF) [file pntd.0002175.s002.pdf]
