# Supplementary material for: Dual African Origins of Global Aedes aegypti s.l. Populations Revealed by Mitochondrial DNA
Source: PLoS Negl Trop Dis. 2013 Apr 18;7(4):e2175. doi: 10.1371/journal.pntd.0002175 (PMC3630099; doi:10.1371/journal.pntd.0002175)
Supplement: Table S1 — Unique mitochondrial ND4 sequences in GenBank (117 total) listed according to the order of appearance in the literature or in GenBank. The third column contains the GenBank # for entries with identical sequences to the initial submission. There are 214 entries (rows) in the table. The 4th column indicates whether the haplotype occurs in the basal (B) or the derived (D) clade. The name of the haplotype as it appears in the original publication (column 7) is listed in the 5th columns and the collection location in the 6th column. (DOC) [file pntd.0002175.s003.doc]

**Table S1.** Unique mitochondrial ND4 sequences in GenBank (117 total) listed according to the order of appearance in the literature or in GenBank. The third column contains the GenBank # for entries with identical sequences to the initial submission. There are 214 entries (rows) in the table. The 4^th^ column indicates whether the haplotype occurs in the basal (B) or the derived (D) clade. The name of the haplotype as it appears in the original publication (column 7) is listed in the 5^th^ columns and the collection location in the 6^th^ column.

| # | GeneBank # | Identical Seq. | Clade | Haplotype Name in Pub | Collection location | Pub* |
| --- | --- | --- | --- | --- | --- | --- |
| 1 | AF203344 |  | D | ND4 1a | Mexico, North America | 23 |
|  |  | AF203345 |  | ND4 1b | Mexico, North America | 23 |
|  |  | AF203354 |  | ND4 5a | Mexico, North America | 23 |
|  |  | AF203355 |  | ND4 5b | Mexico, North America | 23 |
|  |  | AF203357 |  | ND4 7a | Mexico, North America | 23 |
|  |  | AF203359 |  | ND4 7c | Mexico, North America | 23 |
|  |  | AF203360 |  | ND4 8b | Mexico, North America | 23 |
|  |  | AF203361 |  | ND4 9a | Mexico, North America | 23 |
|  |  | AF203362 |  | ND4 9b | Mexico, North America | 23 |
|  |  | AF334841 |  | ND4 isolate 1 | Mexico, North America | 24 |
|  |  | AF334856 |  | ND4 isolate 16 | Mexico, North America | 24 |
|  |  | DQ177154 |  | Piura 1 | Peru, South America | 26 |
|  |  | EF153744 |  | ND4 haplotype 1 | Brazil, South America | GB1 |
|  |  | EF153749 |  | ND4 haplotype 6 | Brazil, South America | GB1 |
|  |  | EF153753 |  | ND4 haplotype 10 | Brazil, South America | GB1 |
|  |  | EF153759 |  | ND4 haplotype 16 | Brazil, South America | GB1 |
|  |  | DQ176828 |  | ND4 haplotype 01 | Brazil/Peru, South America | 28 |
|  |  | AY906835 |  | ND4 clone 1 | Brazil, South America | 30 |
| 2 | AF203346 |  | B | ND4 2a | Mexico, North America | 23 |
|  |  | AF203347 |  | ND4 2b | Mexico, North America | 23 |
|  |  | AF203349 |  | ND4 3b | Mexico, North America | 23 |
|  |  | AF203358 |  | ND4 7b | Mexico, North America | 23 |
|  |  | AF203363 |  | ND4 10b | Mexico, North America | 23 |
|  |  | AF203364 |  | ND4 10a | Mexico, North America | 23 |
|  |  | AF203365 |  | ND4 11a | Mexico, North America | 23 |
|  |  | AF203366 |  | ND4 11b | Mexico, North America | 23 |
|  |  | AF334843 |  | ND4 isolate 3 | Mexico, North America | 24 |
|  |  | AF334844 |  | ND4 isolate 4 | Mexico, North America | 24 |
|  |  | DQ177153 |  | Lima | Peru, South America | 26 |
|  |  | DQ176842 |  | ND4 haplotype 16 | Brazil/Peru/USA, Americas | 28 |
|  |  | EU650409 |  | ND4 H5 | Brazil, South America | 35 |
|  |  | AY906851 |  | ND4 clone 17 | Brazil, South America | 30 |
|  |  | Vene03 |  | Vene03 | Venezuela, South America | 31 |
|  |  | FJ428779 |  | C5 16A | Cambodia, Asia | 33 |
| 3 | AF203348 |  | B | ND4 3a | Mexico, North America | 23 |
|  |  | AF203350 |  | ND4 3c | Mexico, North America | 23 |
|  |  | AF334845 |  | ND4 isolate 5 | Mexico, North America | 24 |
|  |  | ThaiHap5 |  | ThaiHap5 | Thailand, Asia | 25 |
|  |  | DQ176839 |  | ND4 haplotype 13 | Tahiti/Cambodia/Singapore/Brzil | 28 |
|  |  | EU446269 |  | ND4 isolate 6 | Kenya, Eastern Africa | ** |
|  |  | EU446277 |  | ND4 isolate 7 | Kenya, Eastern Africa | ** |
|  |  | EU650407 |  | ND4 H3 | Brazil/Mexico, Americas | 35 |
|  |  | AY906850 |  | ND4 clone 16 | Brazil, South America | 30 |
|  |  | Vene5 |  | Vene5 | Venezuela, South America | 31 |
|  |  | FJ428759 |  | M1_1L | Myanmar, Asia | 33 |
|  |  | FJ428768 |  | T1_22A | Thailand, Asia | 33 |
|  |  | FJ428773 |  | M3_7L | Myanmar, Asia | 33 |
|  |  | FJ428774 |  | T1_8A | Thailand, Asia | 33 |
|  |  | FJ428781 |  | C1_22A | Cambodia, Asia | 33 |
|  |  | FJ428784 |  | T6_3P | Thailand, Asia | 33 |
| 4 | AF203351 |  | D | ND4 4a | Mexico, North America | 23 |
|  |  | AF203352 |  | ND4 4b | Mexico, North America | 23 |
|  |  | AF203353 |  | ND4 4c | Mexico, North America | 23 |
|  |  | AF334842 |  | ND4 isolate 2 | Mexico, North America | 24 |
|  |  | AF334858 |  | ND4 isolate 18 | Mexico, North America | 24 |
| 5 | AF203356 |  | D | ND4 6b | Mexico, North America | 23 |
|  |  | AF334846 |  | ND4 isolate 6 | Mexico, North America | 24 |
|  |  | EF153745 |  | ND4 haplotype 2 | Brazil, South America | GB1 |
|  |  | EF153754 |  | ND4 haplotype 11 | Brazil, South America | GB1 |
|  |  | DQ176833 |  | ND4 haplotype 07 | USA/Brazil/Senegal | 28 |
|  |  | EU650415 |  | ND4 H11 | Brazil, South America | 35 |
|  |  | AY906842 |  | ND4 clone 8 | Brazil, South America | 30 |
|  |  | Vene6 |  | Vene06 | Venezuela, South America | 31 |
|  |  | FJ428790 |  | M4_16A | Myanmar, Asia | 33 |
| 6 | AF334860 |  | D | ND4 isolate 20 | USA, North America | 23 |
|  |  | EF153751 |  | ND4 haplotype 8 | Brazil, South America | GB1 |
|  |  | DQ176831 |  | ND4 haplotype 05 | Brazil, South America | 28 |
|  |  | AY906836 |  | ND4 clone 2 | Brazil, South America | 30 |
| 7 | AF334863 |  | D | ND4 isolate 23 | Mexico, North America | 23 |
|  |  | EF153757 |  | ND4 haplotype 14 | Brazil, South America | GB1 |
|  |  | AY906837 |  | ND4 clone 3 | Brazil, South America | 30 |
| 8 | DQ177155 |  | B | Piura 2 | Peru, South America | 26 |
|  |  | EF153746 |  | ND4 haplotype 3 | Brazil, South America | GB1 |
|  |  | EF153750 |  | ND4 haplotype 7 | Brazil, South America | GB1 |
|  |  | DQ176841 |  | ND4 haplotype 15 | Brazil/Peru, South America | 28 |
|  |  | EU650405 |  | ND4 H1 | Brazil, South America | 35 |
|  |  | JN089748 |  | isolate A | Brazil, South America | GB3 |
| 9 | DQ440244 |  | D | clone AET-3506 | Location Not Specified | 37 |
|  |  | DQ176830 |  | ND4 haplotype 03 | Senegal, Western Africa | 28 |
|  |  |  |  | Vene27 | Venezuela, South America | 31 |
|  |  | FJ428783 |  | T2_64A | Thailand, Asia | 33 |
|  |  | FJ428791 |  | T10_21A | Thailand, Asia | 33 |
| 10 | JX427509 |  | D | ThaiHap31 | Thailand, Asia | 25 |
|  |  | FJ428789 |  | T10_9L | Thailand, Asia | 33 |
| 11 | EF153747 |  | B | ND4 haplotype 4 | Brazil, South America | GB1 |
|  |  | EF153752 |  | ND4 haplotype 9 | Brazil, South America | GB1 |
|  |  | JN190355 |  | foze01b | Brazil, South America | GB2 |
|  |  | DQ176843 |  | ND4 haplotype 17 | Brazil, South America | 28 |
|  |  | EU650410 |  | ND4 H6 | Brazil/Mexico, Americas | 35 |
|  |  | AY906853 |  | ND4 clone 19 | Brazil, South America | 30 |
|  |  | FJ428776 |  | M4_17A | Myanmar, Asia | 33 |
| 12 | EF153755 |  | B | hap 12 | Brazil, South America | GB1 |
|  |  | AY906845 |  | ND4 clone 11 | Brazil, South America | 30 |
| 13 | DQ176834 |  | D | ND4 haplotype 08 | Brazil/Venezuela, South America | 28 |
|  |  |  |  | Vene29 | Venezuela, South America | 31 |
| 14 | DQ176835 |  | D | ND4 haplotype 09 | Brazil/USA, Americas | 28 |
|  |  |  |  | Vene28 | Venezuela, South America | 31 |
| 15 | DQ176837 |  | B | ND4 haplotype 11 | Guinea/Uganda/Singapore | 28 |
|  |  | EF562501 |  | haplotype 1 | Cameroon, Western Africa | 29 |
|  |  | AY906847 |  | ND4 clone 12 | Brazil, South America | 30 |
|  |  | FJ428769 |  | M1_20L | Myanmar, Asia | 33 |
|  |  | Zig39 |  | Zig39 | Senegal, Western Africa | 38 |
| 16 | DQ176845 |  | B | ND4 haplotype 19 | USA, North America | 28 |
|  |  | AY906852 |  | ND4 clone 18 | Brazil, South America | 30 |
| 17 | DQ176848 |  | B | ND4 haplotype 22 | USA, North America | 28 |
|  |  | AY906846 |  | ND4 clone 13 | Brazil, South America | 30 |
| 18 | JN190356 |  | D | voucher foze10l | Brazil, South America | GB2 |
|  |  | AY906839 |  | ND4 clone 5 | Brazil, South America | 30 |
|  |  | JN089751 |  | isolate "D" | Brazil, South America | GB3 |
| 19 | AY906841 |  | D | ND4 clone 07 | Brazil, South America | 30 |
|  |  | EU446270 |  | isolate 11 | Kenya, Eastern Africa | ** |
| 20 | FJ428760 |  | B | T1_1A | Thailand, Asia | 33 |
|  |  | FJ428761 |  | T1_3A | Thailand, Asia | 33 |
|  |  | FJ428762 |  | C5_50A | Cambodia, Asia | 33 |
|  |  | FJ428763 |  | T3_107A | Thailand, Asia | 33 |
|  |  | FJ428764 |  | T3_86A | Thailand, Asia | 33 |
| 21 | FJ428792 |  | D | T4_158A | Thailand, Asia | 33 |
|  |  | FJ428793 |  | T4_147A | Thailand, Asia | 33 |
| 22 | AF203367 |  | B | ND4 12a | Mexico, North America | 23 |
| 23 | AF203368 |  | B | ND4 12b | Mexico, North America | 23 |
| 24 | AF334847 |  | B | ND4 isolate 07 | Mexico, North America | 24 |
| 25 | AF334848 |  | B | ND4 isolate 08 | Mexico, North America | 24 |
| 26 | AF334849 |  | B | ND4 isolate 09 | USA, North America | 24 |
| 27 | AF334850 |  | B | ND4 isolate 10 | Mexico, North America | 24 |
| 28 | AF334851 |  | B | ND4 isolate 11 | Mexico, North America | 24 |
| 29 | AF334852 |  | B | ND4 isolate 12 | Mexico, North America | 24 |
| 30 | AF334853 |  | B | ND4 isolate 13 | Mexico/USA, Americas | 24 |
| 31 | AF334854 |  | B | ND4 isolate 14 | Mexico, North America | 24 |
| 32 | AF334855 |  | B | ND4 isolate 15 | Mexico, North America | 24 |
| 33 | AF334857 |  | D | ND4 isolate 17 | Mexico, North America | 24 |
| 34 | AF334859 |  | D | ND4 isolate 19 | Mexico, North America | 24 |
| 35 | AF334861 |  | D | ND4 isolate 21 | Mexico, North America | 24 |
| 36 | AF334862 |  | D | ND4 isolate 22 | Mexico, North America | 24 |
| 37 | AF334864 |  | D | ND4 isolate 24 | Mexico, North America | 24 |
| 38 | AF334865 |  | D | ND4 isolate 25 | Mexico, North America | 24 |
| 39 | JX427505 |  | D | ThaiHap27 | Thailand, Asia | 25 |
| 40 | JX427506 |  | B | ThaiHap28 | Thailand, Asia | 25 |
| 41 | JX427507 |  | D | ThaiHap29 | Thailand, Asia | 25 |
| 42 | JX427508 |  | D | ThaiHap30 | Thailand, Asia | 25 |
| 43 | JX427510 |  | B | ThaiHap32 | Thailand, Asia | 25 |
| 44 | EF153748 |  | D | ND4 haplotype 05 | Brazil, South America | GB1 |
| 45 | EF153756 |  | D | ND4 haplotype 13 | Brazil, South America | GB1 |
| 46 | EF153758 |  | D | ND4 haplotype 15 | Brazil, South America | GB1 |
| 47 | EF153760 |  | B | ND4 haplotype 17 | Brazil, South America | GB1 |
| 48 | DQ176829 |  | D | ND4 haplotype 02 | Brazil, South America | 28 |
| 49 | DQ176836 |  | D | ND4 haplotype 10 | Dakar Senegal, Western Africa | 28 |
| 50 | DQ176838 |  | B | ND4 haplotype 12 | Uganda, Eastern Africa | 28 |
| 51 | DQ176840 |  | B | ND4 haplotype 14 | Brazil, South America | 28 |
| 52 | DQ176846 |  | B | ND4 haplotype 20 | Brazil, South America | 28 |
| 53 | DQ176847 |  | B | ND4 haplotype 21 | Brazil, South America | 28 |
| 54 | DQ176849 |  | B | ND4 haplotype 23 | Brazil, South America | 28 |
| 55 | EF562502 |  | B | haplotype 2 | Cameroon, Western Africa | 29 |
| 56 | EF562503 |  | B | haplotype 3 | Cameroon, Western Africa | 29 |
| 57 | EF562504 |  | D | haplotype 4 | Cameroon, Western Africa | 29 |
| 58 | AY906838 |  | D | ND4 clone 04 | Brazil, South America | 30 |
| 59 | AY906840 |  | D | ND4 clone 06 | Brazil, South America | 30 |
| 60 | AY906843 |  | D | ND4 clone 09 | Brazil, South America | 30 |
| 61 | AY906844 |  | B | ND4 clone 10 | Brazil, South America | 30 |
| 62 | AY906848 |  | B | ND4 clone 14 | Brazil, South America | 30 |
| 63 | AY906849 |  | B | ND4 clone 15 | Brazil, South America | 30 |
| 64 | EU446267 |  | D | isolate 02 | Kenya, Eastern Africa | ** |
| 65 | EU446268 |  | D | isolate 13 | Kenya, Eastern Africa | ** |
| 66 | EU446271 |  | D | isolate 08 | Kenya, Eastern Africa | ** |
| 67 | EU446273 |  | D | isolate 09 | Kenya, Eastern Africa | ** |
| 68 | EU446275 |  | D | isolate 03 | Kenya, Eastern Africa | ** |
| 69 | EU446276 |  | D | isolate 12 | Kenya, Eastern Africa | ** |
| 70 | EU446278 |  | B | isolate 10 | Kenya, Eastern Africa | ** |
| 71 | EU650406 |  | B | ND4 H02 | Brazil, South America | 35 |
| 72 | EU650408 |  | B | ND4 H04 | Brazil, South America | 35 |
| 73 | EU650411 |  | D | ND4 H07 | Brazil/Senegal/USA | 35 |
| 74 | EU650412 |  | D | ND4 H08 | Brazil, South America | 35 |
| 75 | EU650413 |  | D | ND4 H09 | Brazil, South America | 35 |
| 76 | EU650414 |  | D | ND4 H10 | Brazil, South America | 35 |
| 77 | EU650416 |  | D | ND4 H12 | Brazil, South America | 35 |
| 78 | EU650417 |  | D | ND4 H13 | Brazil/Senegal/USA | 35 |
| 79 | FJ428765 |  | B | M3_5A | Myanmar, Asia | 33 |
| 80 | FJ428766 |  | B | M2_9L | Myanmar, Asia | 33 |
| 81 | FJ428767 |  | B | M3_10L | Myanmar, Asia | 33 |
| 82 | FJ428770 |  | D | T10_40L | Thailand, Asia | 33 |
| 83 | FJ428771 |  | B | M2_8L | Myanmar, Asia | 33 |
| 84 | FJ428772 |  | B | M1_15L | Myanmar, Asia | 33 |
| 85 | FJ428775 |  | B | M3_1L | Myanmar, Asia | 33 |
| 86 | FJ428777 |  | B | M1_8L | Myanmar, Asia | 33 |
| 87 | FJ428778 |  | B | C1_50A | Cambodia, Asia | 33 |
| 88 | FJ428780 |  | B | T9_10L | Thailand, Asia | 33 |
| 89 | FJ428782 |  | B | M1_7L | Myanmar, Asia | 33 |
| 90 | FJ428785 |  | D | M1_4A | Myanmar, Asia | 33 |
| 91 | FJ428786 |  | D | M4_20A | Myanmar, Asia | 33 |
| 92 | FJ428787 |  | D | M2_6A | Myanmar, Asia | 33 |
| 93 | FJ428788 |  | D | M2_7A | Myanmar, Asia | 33 |
| 94 | FJ428794 |  | D | T3_95A | Thailand, Asia | 33 |
| 95 | FJ428795 |  | D | T6_23P | Thailand, Asia | 33 |
| 96 | FJ428796 |  | D | T5_12L | Thailand, Asia | 33 |
| 97 | JN089749 |  | D | iso B | Brazil, South America | GB3 |
| 98 | JN089750 |  | B | iso C | Brazil, South America | GB3 |
| 99 | JN089752 |  | B | iso E | Brazil, South America | GB3 |
| 100 | JN089753 |  | B | iso F | Brazil, South America | GB3 |
| 101 | JN089754 |  | D | iso G | Brazil, South America | GB3 |
| 102 | JN089755 |  | B | iso H | Brazil, South America | GB3 |
| 103 | JX427511 |  | B | Fg1057 | Senegal, Western Africa | * |
| 104 | JX427512 |  | B | Fg1080 | Senegal, Western Africa | * |
| 105 | JX427513 |  | B | Fg1088 | Senegal, Western Africa | * |
| 106 | JX427514 |  | B | Kn565 | Senegal, Western Africa | * |
| 107 | JX427515 |  | B | Kn594 | Senegal, Western Africa | * |
| 108 | JX427516 |  | B | MR21 | Senegal, Western Africa | * |
| 109 | JX427517 |  | B | MR45 | Senegal, Western Africa | * |
| 110 | JX427518 |  | B | PK116 | Senegal, Western Africa | * |
| 111 | JX427519 |  | B | PK119 | Senegal, Western Africa | * |
| 112 | JX427520 |  | B | PK121 | Senegal, Western Africa | * |
| 113 | JX427521 |  | B | Q06 | Senegal, Western Africa | * |
| 114 | JX427522 |  | B | Q12 | Senegal, Western Africa | * |
| 115 | JX427523 |  | B | Sed45 | Senegal, Western Africa | * |
| 116 | JX427524 |  | B | Zig21 | Senegal, Western Africa | * |
| 117 | JX427525 |  | B | Zig37 | Senegal, Western Africa | * |
