# Supplementary material for: Dual African Origins of Global Aedes aegypti s.l. Populations Revealed by Mitochondrial DNA
Source: PLoS Negl Trop Dis. 2013 Apr 18;7(4):e2175. doi: 10.1371/journal.pntd.0002175 (PMC3630099; doi:10.1371/journal.pntd.0002175)
Supplement: Figure S1 — Maximum likelihood tree of the 117 mtDNA ND4 Ae. aegypti haplotypes discovered to date and outgroups. Haplotypes collected only in Africa appear in red, South America in blue, North America in gold, Southeast Asia in purple and those collected in more than one geographic area in green. All of the NUMTS found in GenBank to date appear as olive drab and are labeled NUMT. Branches with bootstrap support values >50% are labeled with % support. Maximum likelihood branch lengths are proportional to the number of nucleotide substitutions per site. The scale at the bottom of this figure is from 0.0–0.07. (PDF) [file pntd.0002175.s001.pdf]

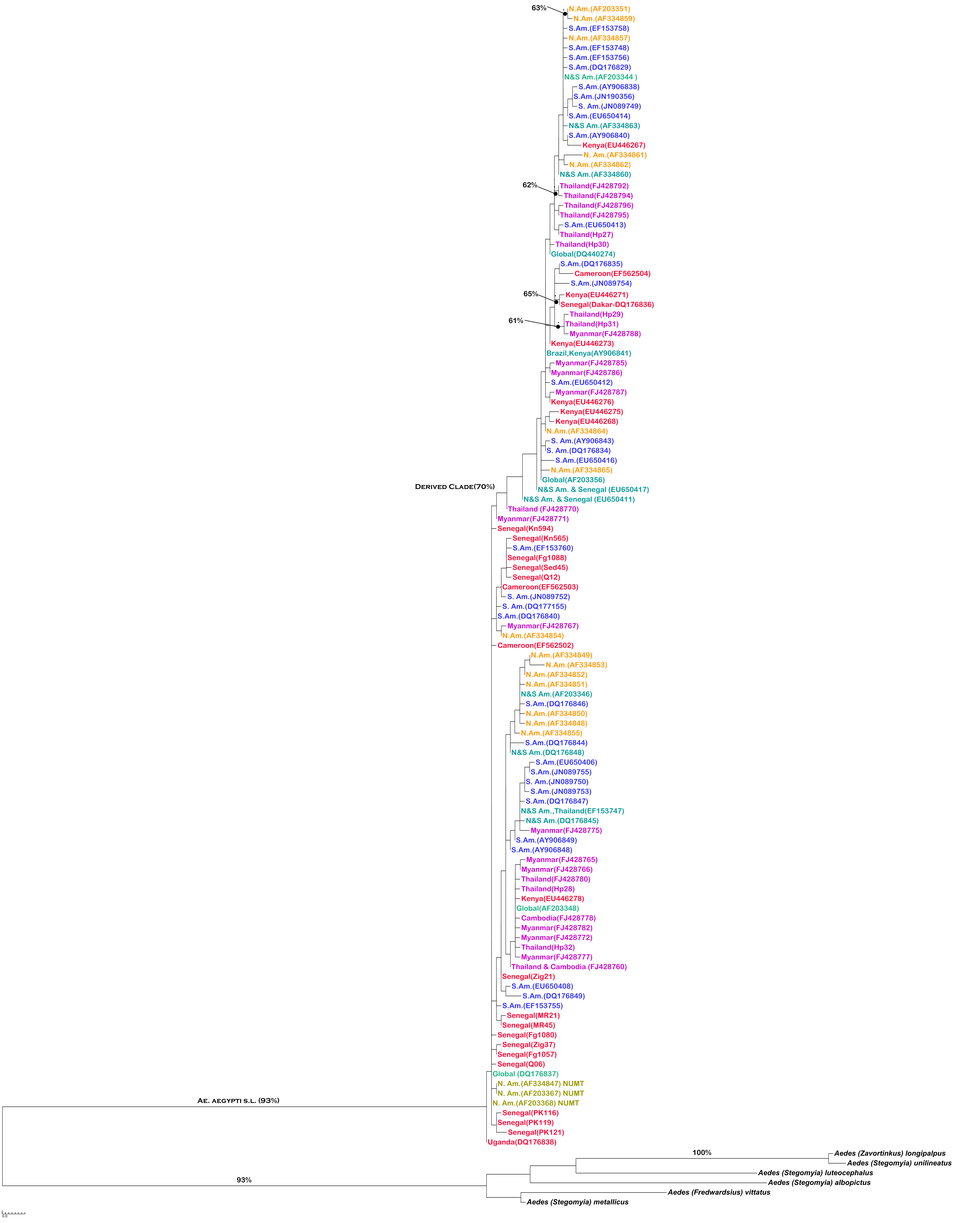
63%
N.Am.(AF203351)
N.Am.(AF334859)
S.Am.(EF153758)
N.Am.(AF334857)
S.Am.(EF153748)
S.Am.(EF153756)
S.Am.(DQ176829)
N&S Am.(AF203344)
S.Am.(AY906838)
S.Am.(JN190356)
S. Am.(JN089749)
S.Am.(EU650414)
N&S Am.(AF334863)
S.Am.(AY906840)
Kenya(EU446267)
N. Am.(AF334861)
N.Am.(AF334862)
N&S Am.(AF334860)
62%
Thailand(FJ428792)
Thailand(FJ428794)
Thailand(FJ428796)
Thailand(FJ428795)
S.Am.(EU650413)
Thailand(Hp27)
Thailand(Hp30)
Global(DQ440274)
S.Am.(DQ176835)
Cameroon(EF562504)
S.Am.(JN089754)
65%
Kenya(EU446271)
Senegal(Dakar-DQ176836)
Thailand(Hp29)
61%
Thailand(Hp31)
Myanmar(FJ428788)
Kenya(EU446273)
Brazil,Kenya(AY906841)
Myanmar(FJ428785)
Myanmar(FJ428786)
S.Am.(EU650412)
Myanmar(FJ428787)
Kenya(EU446276)
Kenya(EU446275)
Kenya(EU446268)
N.Am.(AF334864)
S. Am.(AY906843)
S. Am.(DQ176834)
S.Am.(EU650416)
N.Am.(AF334865)
Global(AF203356)
Derived Clade(70%)
N&S Am. & Senegal (EU650417)
N&S Am. & Senegal (EU650411)
Thailand (FJ428770)
Myanmar(FJ428771)
Senegal(Kn594)
Senegal(Kn565)
S.Am.(EF153760)
Senegal(Fg1088)
Senegal(Sed45)
Senegal(Q12)
Cameroon(EF562503)
S. Am.(JN089752)
S. Am.(DQ177155)
S.Am.(DQ176840)
Myanmar(FJ428767)
N.Am.(AF334854)
Cameroon(EF562502)
N.Am.(AF334849)
N.Am.(AF334853)
N.Am.(AF334852)
N.Am.(AF334851)
N&S Am.(AF203346)
S.Am.(DQ176846)
N.Am.(AF334850)
N.Am.(AF334848)
N.Am.(AF334855)
S.Am.(DQ176844)
N&S Am.(DQ176848)
S.Am.(EU650406)
S.Am.(JN089755)
S. Am.(JN089750)
S.Am.(JN089753)
S.Am.(DQ176847)
N&S Am.,Thailand(EF153747)
N&S Am.(DQ176845)
Myanmar(FJ428775)
S.Am.(AY906849)
S.Am.(AY906848)
Myanmar(FJ428765)
Myanmar(FJ428766)
Thailand(FJ428780)
Thailand(Hp28)
Kenya(EU446278)
Global(AF203348)
Cambodia(FJ428778)
Myanmar(FJ428782)
Myanmar(FJ428772)
Thailand(Hp32)
Myanmar(FJ428777)
Thailand & Cambodia (FJ428760)
Senegal(Zig21)
S.Am.(EU650408)
S.Am.(DQ176849)
S.Am.(EF153755)
Senegal(MR21)
Senegal(MR45)
Senegal(Fg1080)
Senegal(Zig37)
Senegal(Fg1057)
Senegal(Q06)
Global (DQ176837)
N. Am.(AF334847) NUMT
N. Am.(AF203367) NUMT
N. Am.(AF203368) NUMT
Ae. aegypti s.l. (93%)
Senegal(PK116)
Senegal(PK119)
Senegal(PK121)
Uganda(DQ176838)
100%
Aedes (Zavortinkus) longipalpus
Aedes (Stegomyia) unilineatus
Aedes (Stegomyia) luteocephalus
Aedes (Stegomyia) albopictus
93%
Aedes (Fredwardsius) vittatus
Aedes (Stegomyia) metallicus
